# The Revision of the British Pharmacopœia

**Published:** 1909-04-24

**Authors:** 


### Therapeutics and Pharmacy.

### THE REVISION OF THE BRITISH PHARMACOPŒIA.

The Report of the Committee of Reference in Pharmacy to the Pharmacopœia Committee of the General Medical Council embodies the results of work accomplished in connection with the revision of the British Pharmacopœia up to October 29, 1908. It is a pamphlet* of but thirty-two pages; but a perusal of it shows that much unrecognised work is being done for the profession at large. All the various tests that different drugs have to comply with in order to be passed as suitable for medicinal use are being re-tested; some are found too stringent, some inadequate, so that new tests, or modifications in the old ones, have to be devised, and in their turn tested. All this means long and patient work. The profession receives the results passively, scarcely realising how important the work is, and seldom knowing or caring who is responsible for it.

The Committee appears to have directed very special attention to the question of how to detect lead as an impurity in drugs; and the details of the quantitative colorimetric lead test that is regarded as the best are given in an appendix. Rules are laid down as to the maximum quantity of lead permissible in various preparations. The proportions of lead suggested as allowable vary from none at all to 20 parts per million.

The Revising Committee, except in so far as lead is concerned, is taking the Pharmacopœia alphabetically, and when its report was published it had reached *extractum gentianæ*. Amongst the large numbers of points dealt with the following may be mentioned: The old ferric chloride test for acetanilide should be omitted, as the reaction occurs even when acetanilide is not present. The following formula for acetum cantharidis was experimentally confirmed and agreed to, the name to be altered to acetum cantharidini:

| | |
|---|---|
| Cantharidin ... ... ... ... | 1 gm. |
| Glacial acetic acid ... ... ... | 200 c.c. |
| Acetic acid ... ... ... ... ... | 2,000 c.c. |

This preparation will be of definite strength, and yet more easily made than the present official acetum. Acidum carbolicum liquefactum is recommended to be made by adding sufficient water to each 50 grains of phenol to make it up to one fluid drachm; this would be slightly weaker than the present preparation, but it would be of constant strength, and the additional water thus introduced would be useful in preventing crystallisation of the acid. The best means of detecting cotton seed oil in the lard used for ointment is engaging the attention of the Committee, and experiments are being carried out on this point.

In view of recent discussions in regard to anæsthetics the remarks upon ether are important. It is recommended that the description of its production should read "may be prepared." This modification is desirable to permit the use of ether prepared in this country from industrial alcohol; such ether can be and is prepared on the commercial scale of such purity as to be indistinguishable from ether made from dutiable alcohol. To exclude the presence of methyl oxide it should boil at a temperature not lower than 34° C. Under the name of "Æther anæstheticus" an ether for anæsthetic purposes with more stringent tests should be introduced. Experiments having shown that the solid potash test of the German Pharmacopœia for ether pro narcosi is too stringent, it might be replaced by the following: "If caustic potash in small fragments be kept in contact with the ether in a well-stoppered bottle protected from the light, no yellow colouration should be developed within one hour."

In regard to aloes it is recommended that the epithets Barbadensis and Socotrina be no longer used, the drug being described simply as aloes. The object of this is to discourage the use of Socotrine and Zanzibar aloes, and to encourage that of the better prepared Curaçoa (Barbados) variety, though either variety could still be used provided the product comes up to the standards laid down in the recommendation. Experiments are being carried out with amyl nitrite with a view to improving the description and tests applicable to it. Aqua destillata receives considerable attention; it may be thought that one distilled water is as good as another, but this is by no means the case, especially as regards its alkalinity and ammonia content. The recommendations made in regard to it are stringent.

Synthetic camphor has come into the market since the last revision of the Pharmacopœia, but it is recommended that it should not be officially recognised at present, as it is not identical with natural camphor. In order to exclude the synthetic variety the requirements should be made that camphor should melt at 175° C., and that a solution of 25 gm. in 90 per cent. alcohol, sufficient to produce 100 c.c. at 16° C., should exhibit an optical rotation of about +10° when examined in a tube 100 mm. long. Many practitioners would be sorry to see Charta Sinapis disappear from the Pharmacopœia; but it seems that it is possible that it may do so, for the Committee report that "if this preparation is retained the formula must be revised."

In regard to chloroformum it is recommended that as chloroform to which 2 per cent. of ethylic alcohol has been added keeps indefinitely and under all conditions, this addition should be made. The specific gravity and boiling point of this mixture are being determined.

Colchicum seeds vary so much in toxicity that it is thought advisable to standardise them. Standardisation would be by estimation of the colchicine present, and a method of determining this active principle is given in full. As an improvement upon the two collodions that are at present official, the following formula is recommended:

| | |
|---|---|
| Pyroxylin ... ... ... ... | 5 parts. |
| Oil of cloves ... ... ... ... | 2 ,, |
| Amyl acetate ... ... ... | 25 ,, |
| Benzene ... ... ... ... | 20 ,, |
| Acetone to produce ... ... | 100 ,, |

* Printed, published, and sold for the Medical Council by Spottiswoode and Co., Ltd., London.

